# Evaluating Artificial Intelligence's Role in Teaching the Reporting and Interpretation of Computed Tomographic Angiography for Preoperative Planning of the Deep Inferior Epigastric Artery Perforator Flap

**DOI:** 10.1016/j.jpra.2024.03.010

**Published:** 2024-04-05

**Authors:** Bryan Lim, Jevan Cevik, Ishith Seth, Foti Sofiadellis, Richard J. Ross, Warren M. Rozen, Roberto Cuomo

**Affiliations:** a Department of Plastic Surgery, Peninsula Health, Melbourne, Victoria, 3199, Australia; b Faculty of Medicine, Monash University, Melbourne, Victoria, 3004, Australia; c Plastic Surgery Unit, Department of Medicine, Surgery and Neuroscience, University of Siena, 53100, Italy

**Keywords:** CT Angiogram, CTA, Large Language Models, ChatGPT, BARD, Bing

## Abstract

**Background:**

Artificial intelligence (AI) has the potential to transform preoperative planning for breast reconstruction by enhancing the efficiency, accuracy, and reliability of radiology reporting through automatic interpretation and perforator identification. Large language models (LLMs) have recently advanced significantly in medicine. This study aimed to evaluate the proficiency of contemporary LLMs in interpreting computed tomography angiography (CTA) scans for deep inferior epigastric perforator (DIEP) flap preoperative planning.

**Methods:**

Four prominent LLMs, ChatGPT-4, BARD, Perplexity, and BingAI, answered six questions on CTA scan reporting. A panel of expert plastic surgeons with extensive experience in breast reconstruction assessed the responses using a Likert scale. In contrast, the responses’ readability was evaluated using the Flesch Reading Ease score, the Flesch-Kincaid Grade level, and the Coleman-Liau Index. The DISCERN score was utilized to determine the responses’ suitability. Statistical significance was identified through a t-test, and P-values < 0.05 were considered significant.

**Results:**

BingAI provided the most accurate and useful responses to prompts, followed by Perplexity, ChatGPT, and then BARD. BingAI had the greatest Flesh Reading Ease (34.7±5.5) and DISCERN (60.5±3.9) scores. Perplexity had higher Flesch-Kincaid Grade level (20.5±2.7) and Coleman-Liau Index (17.8±1.6) scores than other LLMs.

**Conclusion:**

LLMs exhibit limitations in their capabilities of reporting CTA for preoperative planning of breast reconstruction, yet the rapid advancements in technology hint at a promising future. AI stands poised to enhance the education of CTA reporting and aid preoperative planning. In the future, AI technology could provide automatic CTA interpretation, enhancing the efficiency, accuracy, and reliability of CTA reports.

## Introduction

The progressive development of artificial intelligence (AI), particularly in relation to automated computed tomography angiogram (CTA) reporting, brings unprecedented possibilities to the forefront, especially when considering large language models' (LLMs') capacity to emulate human-like textual outputs.^1^^,^^2^ This advancement underscores new horizons for medical education, offering potential benefits to radiology and plastic surgery trainees that may positively impact patient outcomes. Nevertheless, the full potential of LLMs in specialized tasks like radiological interpretation is yet to be thoroughly explored.

CTA advancements have occurred in radiology with machine learning (ML), but their integration in preoperative planning for surgical procedures remains underdeveloped.^3^^,^^4^ Deep inferior epigastric perforator (DIEP) flap surgeries are challenging due to variations in location, size, and number of perforators.^5^, ^6^, ^7^, ^8^ CTA scans are often employed preoperatively to map these perforators as they optimize surgery time and decision-making.^6^^,^^9^, ^10^, ^11^, ^12^ Accurately assessing the deep inferior epigastric artery (DIEA) and associated anatomical structures is crucial for optimal preoperative planning.^12^

Traditionally, CTA scans are reported manually by surgeons or radiologists, and several postprocessing procedures are used to generate comprehensive three-dimensional (3D) reconstructions of the abdominal wall and its vasculature. The locations of key anatomical structures are indicated, and a report is generated for preoperative visualization. This area suffers from a paucity of standardized reporting guidelines, limited learning resources, inadequate instruction, and suboptimal AI-savviness by professionals, all of which hinder the education of this reporting process, particularly affecting new trainees.^13^^,^^14^ Given the potential benefits of AI technology, it is hypothesized that they could offer a novel adjunct in the educational process of reporting CTA scans for preoperative planning in breast reconstruction.

**Figure 1:**
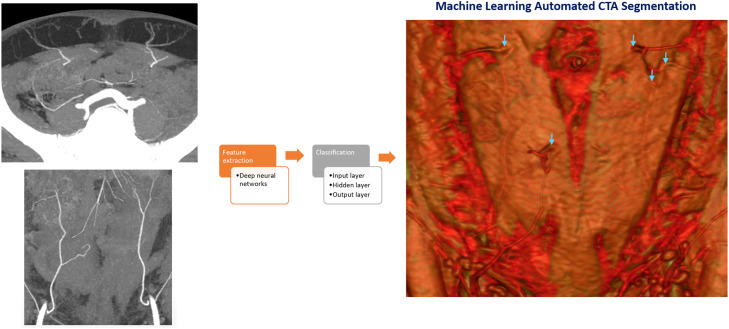
Use of machine learning properties of computational models and algorithms to automatically conduct computed tomography angiogram segmentation.

In addition, AI can revolutionize preoperative planning for breast reconstruction by enhancing the interpretation of DIEA CTA scans. After ML algorithms detect anatomical features in images, they generate and adjust random parameters based on prediction errors, enabling them to predict novel images^15^ (Figure 1). As a result, the amalgamation of AI and ML, as seen in LLMs, in CTA reporting can significantly improve the efficiency, accuracy, and reliability of preoperative planning, offering a more streamlined and precise approach to surgical procedures.

This study scrutinized the accuracy and effectiveness of four LLMs, ChatGPT-4, BARD, BingAI, and Perplexity AI, in providing accurate information on DIEA CTA interpretation. Additionally, this study secondarily aimed to assess the current understanding of modern LLMs in the preoperative planning of autologous breast reconstruction and identify areas for improvement, an area not extensively covered in the current literature.

## Materials and Methods

Four LLMs—ChatGPT-4, BARD, BingAI, and Perplexity—were prompted with six questions designed by three experienced plastic surgeons to assess LLMs knowledge depth, breadth, and their explanatory ability on CTA scans for breast reconstruction (Supplementary Files 1-4), and example of LLMs user interface of prompts is seen in Supplementary Figure 1. All prompts were inputted into the three LLMs in one sitting by one author (BL) to reduce potential variations caused by different users or sessions. The LLMs’ outputs were compared with standard DIEA CTA scanning parameters (Table 1) and requested to include five high-quality references, which were subsequently verified against databases like PubMed, Scopus, EMBASE, and Cochrane CENTRAL. Institutional ethics approval was not required. Only the first response was included to ensure fair comparison and was evaluated by Specialist Plastic Surgeons with extensive experience in breast reconstruction using a Likert scale (Table 2).

**Table 1:** CTA Scanning Parameters and Post-processing Reporting Protocol for DIEP Preoperative Planning.

**Scanner: GE REVOLUTION 256 SLICE** (GE Healthcare, Milwaukee, Wisconsin, USA)
Slice Thickness: 0.625mm
Detector Pitch: 0.992
Gantry Rotation Speed: 0.5s
Tube Potential: 100–120 kV (depending on patient weight/size)
Tube Current: 100–350 mA (Smart mA)
IV Contrast: 100mL of Omnipaque 350 at a rate of 4.0–5.0 mL/s
Range: Lesser Trochanter to L2
Bolus tracking: Scan when contrast present (+100HU) in common femoral artery
Post Processing: • Images downloaded in DICOM format from local Picture Archiving and Communication System (PACS) • Rendered in processing software Horos^TM^ (The Horos Project, Nimble Co LLC Purview, Annapolis, MD, USA). • Intramuscular, subfascial, and subcutaneous course of perforators assessed in axial plane using MIP • DIEA pedicle type and course assessed in the sagittal plane using MIP • 3D reconstruction generated using volume rendering technique (VRT) and specific color look-up table (CLUT) • VRT 3D reconstruction processed to remove overlying abdominal skin and subcutaneous fat is made transparent to reveal perforating vessels • Perforator size, location (where they pierce rectus sheath relative to umbilicus), and number assessed on MIP and VRT images and annotated on images provided to surgeons • SIEA/SIEV anatomy assessed and described in a report • Abdominal wall structure and presence of any pertinent defects analyzed and mentioned in the report

**Table 2:** Evaluation of large language model platforms' responses.

Criteria	ChatGPT	BingAI	Google's BARD	Perplexity
**The large language model provides accurate answers to questions.**	[] 1 – Strongly Disagree [] 2 – Disagree [x] 3 – Neither Agree or Disagree []4 – Agree [] 5 – Strongly Agree	[] 1 – Strongly Disagree [] 2 – Disagree [] 3 – Neither Agree or Disagree [x] 4 – Agree [] 5 – Strongly Agree	[] 1 – Strongly Disagree [] 2 – Disagree [x] 3 – Neither Agree or Disagree [] 4 – Agree [] 5 – Strongly Agree	[] 1 – Strongly Disagree [] 2 – Disagree [] 3 – Neither Agree or Disagree [x] 4 – Agree [] 5 – Strongly Agree
**The large language model is reliable when generating factual information.**	[] 1 – Strongly Disagree [] 2 – Disagree [x] 3 – Neither Agree or Disagree [] 4 – Agree [] 5 – Strongly Agree	[] 1 – Strongly Disagree [] 2 – Disagree [] 3 – Neither Agree or Disagree [x] 4 – Agree [] 5 – Strongly Agree	[] 1 – Strongly Disagree [] 2 – Disagree [x] 3 – Neither Agree or Disagree [] 4 – Agree [] 5 – Strongly Agree	[] 1 – Strongly Disagree [] 2 – Disagree [] 3 – Neither Agree or Disagree [x] 4 – Agree [] 5 – Strongly Agree
**The large language model is proficient at understanding complex questions and providing appropriate answers.**	[] 1 – Strongly Disagree [x] 2 – Disagree [] 3 – Neither Agree or Disagree [] 4 – Agree [] 5 – Strongly Agree	[] 1 – Strongly Disagree [] 2 – Disagree [x] 3 – Neither Agree or Disagree [] 4 – Agree [] 5 – Strongly Agree	[] 1 – Strongly Disagree [x] 2 – Disagree [] 3 – Neither Agree or Disagree [] 4 – Agree [] 5 – Strongly Agree	[] 1 – Strongly Disagree [x] 2 – Disagree [] 3 – Neither Agree or Disagree [] 4 – Agree [] 5 – Strongly Agree
**The large language model provides comprehensive information when answering questions.**	[] 1 – Strongly Disagree [] 2 – Disagree [x] 3 – Neither Agree or Disagree [] 4 – Agree [] 5 – Strongly Agree	[] 1 – Strongly Disagree [] 2 – Disagree [] 3 – Neither Agree or Disagree [x] 4 – Agree [] 5 – Strongly Agree	[] 1 – Strongly Disagree [x] 2 – Disagree [] 3 – Neither Agree or Disagree [] 4 – Agree [] 5 – Strongly Agree	[] 1 – Strongly Disagree [] 2 – Disagree [x] 3 – Neither Agree or Disagree [] 4 – Agree [] 5 – Strongly Agree
**The large language model generates content that covers all relevant aspects of a subject.**	[] 1 – Strongly Disagree [x] 2 – Disagree [] 3 – Neither Agree or Disagree [] 4 – Agree [] 5 – Strongly Agree	[] 1 – Strongly Disagree [] 2 – Disagree [x] 3 – Neither Agree or Disagree [] 4 – Agree [] 5 – Strongly Agree	[] 1 – Strongly Disagree [x] 2 – Disagree [] 3 – Neither Agree or Disagree [] 4 – Agree [] 5 – Strongly Agree	[] 1 – Strongly Disagree [x] 2 – Disagree [] 3 – Neither Agree or Disagree [] 4 – Agree [] 5 – Strongly Agree
**The large language model is able to provide in-depth information on a wide range of topics.**	[] 1 – Strongly Disagree [x] 2 – Disagree [] 3 – Neither Agree or Disagree [] 4 – Agree [] 5 – Strongly Agree	[] 1 – Strongly Disagree [x] 2 – Disagree [] 3 – Neither Agree or Disagree [x] 4 – Agree [] 5 – Strongly Agree	[] 1 – Strongly Disagree [x] 2 – Disagree [] 3 – Neither Agree or Disagree [] 4 – Agree [] 5 – Strongly Agree	[] 1 – Strongly Disagree [x] 2 – Disagree [] 3 – Neither Agree or Disagree [] 4 – Agree [] 5 – Strongly Agree
**The large language model is a valuable source of general knowledge.**	[] 1 – Strongly Disagree [] 2 – Disagree [] 3 – Neither Agree or Disagree [x] 4 – Agree [] 5 – Strongly Agree	[] 1 – Strongly Disagree [] 2 – Disagree [] 3 – Neither Agree or Disagree [] 4 – Agree [x] 5 – Strongly Agree	[] 1 – Strongly Disagree [] 2 – Disagree [] 3 – Neither Agree or Disagree [x] 4 – Agree [] 5 – Strongly Agree	[] 1 – Strongly Disagree [] 2 – Disagree [] 3 – Neither Agree or Disagree [x] 4 – Agree [] 5 – Strongly Agree
**The large language model is well-versed in a variety of subjects.**	[] 1 – Strongly Disagree [] 2 – Disagree [] 3 – Neither Agree or Disagree [x] 4 – Agree [] 5 – Strongly Agree	[] 1 – Strongly Disagree [] 2 – Disagree [] 3 – Neither Agree or Disagree [x] 4 – Agree [] 5 – Strongly Agree	[] 1 – Strongly Disagree [] 2 – Disagree [x] 3 – Neither Agree or Disagree [] 4 – Agree [] 5 – Strongly Agree	[] 1 – Strongly Disagree [] 2 – Disagree [x] 3 – Neither Agree or Disagree [] 4 – Agree [] 5 – Strongly Agree
**The large language model can provide useful insights and perspectives on various topics.**	[] 1 – Strongly Disagree [] 2 – Disagree [] 3 – Neither Agree or Disagree [x] 4 – Agree [] 5 – Strongly Agree	[] 1 – Strongly Disagree [x] 2 – Disagree [] 3 – Neither Agree or Disagree [] 4 – Agree [] 5 – Strongly Agree	[] 1 – Strongly Disagree [] 2 – Disagree [x] 3 – Neither Agree or Disagree [] 4 – Agree [] 5 – Strongly Agree	[] 1 – Strongly Disagree [] 2 – Disagree [] 3 – Neither Agree or Disagree [x] 4 – Agree [] 5 – Strongly Agree
**The large language model rarely makes errors when referencing sources.**	[] 1 – Strongly Disagree [x] 2 – Disagree [] 3 – Neither Agree or Disagree [] 4 – Agree [] 5 – Strongly Agree	[] 1 – Strongly Disagree [] 2 – Disagree [] 3 – Neither Agree or Disagree [x] 4 – Agree [] 5 – Strongly Agree	[x] 1 – Strongly Disagree [] 2 – Disagree [] 3 – Neither Agree or Disagree [] 4 – Agree [] 5 – Strongly Agree	[] 1 – Strongly Disagree [] 2 – Disagree [] 3 – Neither Agree or Disagree [x] 4 – Agree [] 5 – Strongly Agree
**The large language model is consistent in providing accurate citations.**	[] 1 – Strongly Disagree [x] 2 – Disagree [] 3 – Neither Agree or Disagree [] 4 – Agree [] 5 – Strongly Agree	[] 1 – Strongly Disagree [] 2 – Disagree [] 3 – Neither Agree or Disagree [x] 4 – Agree [] 5 – Strongly Agree	[x] 1 – Strongly Disagree [] 2 – Disagree [] 3 – Neither Agree or Disagree [] 4 – Agree [] 5 – Strongly Agree	[] 1 – Strongly Disagree [] 2 – Disagree [x] 3 – Neither Agree or Disagree [] 4 – Agree [] 5 – Strongly Agree

The readability of the responses was assessed using the Flesch Reading Ease score (range -100, with a higher score indicating easier readability), Flesch-Kincaid Grade level, and Coleman-Liau Index (both have no theoretical upper limits, lower scores indicate simpler texts), whereas the DISCERN score (range 16-80, higher scores mean greater quality) was used to evaluate the suitability of the response in conveying information. For statistical analysis, a t-test was applied to analyze the differences between the four LLMs, and a P-value < 0.05 was considered statistically significant.

## Results

### Prompt 1

In response to the first query (Supplementary Figure 1 and Supplementary Files 1-4), BARD identified five factors but only elucidated the intramuscular course, missing key steps such as assessing the superficial inferior epigastric artery (SIEA), superficial inferior epigastric veins (SIEV) and the importance of 3D reconstructions, volume rendering techniques (VRT) or maximum intensity projections (MIP). All five references were unverifiable in the literature. BingAI offered seven factors, introducing the SIEA, SIEV, and abdominal wall structures. It provided comprehensive explanations for each factor, with four of five references being verifiable. Although it provided a good summary of the structures to include in a report, it omitted key procedural steps such as using VRT for 3D reconstruction or MIP for assessment of the perforator intramuscular course.

In contrast, ChatGPT provided a generalized overview but lacked significant detail. It alerted users of potential advancements past September 2021, implying its training data may be outdated and should be taken with caution. Of its references, the third had an incorrect publication date, and the last two were uncorroborated. Perplexity delineated a systematic five-step approach but omitted discussion on VRT or MIP images. It underscored institutional protocols, surgeon preferences, and the importance of contextual factors in decision-making. All its cited references were valid and exist within the current literature, and it supplemented its response by offering additional readings. Overall, all models lacked sufficient detail regarding the importance of generating 3D reconstructions using VRT and assessment of the intramuscular course of perforators/DIEA pedicles using MIP.

### Prompt 2

Key anatomical considerations when reporting CTA scans for preoperative planning of DIEP flaps have been previously reported^23^^,^^24^ and include the following:

1. Perforator size and location
2. Perforator angiosome
3. Intramuscular course
4. DIEA pedicle
5. Venous anatomy
6. SIEA and SIEV
7. Abdominal wall structure

In evaluating the LLMs’ capabilities to identify pertinent structures in a CTA for DIEP flaps, BARD noted five of the above factors but failed to discuss their significance or include perforator angiosomes or abdominal wall structure (Supplementary Files 1-4). Furthermore, its citations remained identical and unverifiable compared to its initial response. BingAI concisely explained six relevant factors, delving into DIEA pedicle types, which affect the intramuscular distance of its perforators. Similarly, it highlighted the SIEA's importance as an alternative blood supply, the SIEV's alternative venous drainage pathway, and identifying structural abnormalities in the abdominal wall. Furthermore, all its references were verifiable. However, it failed to discuss perforator angiosomes. ChatGPT gave a poor response, lacking key anatomical structures such as the perforator angiosomes, DIEA pedicle, SIEA, and abdominal wall structure. Its second and third references had incorrect publication dates, whereas the first and fifth were unverifiable. Perplexity included assessment of skin and subcutaneous tissue; however, this is typically done during physical examination rather than in radiological reports. Additionally, it failed to clarify each component's importance. Nevertheless, the five references it provides were valid.

### Prompt 3

The third query (Supplementary Files 1-4) assessed the LLMs’ proficiencies in identifying vital images for the preoperative planning of DIEP flaps, including MIP axial and coronal/sagittal sections displaying perforator intramuscular course and DIEA/pedicles respectively, and 3D abdominal wall reconstructions using multiplanar reconstruction (MPR) or VRT and an appropriate color lookup tool to display perforators visually. Annotations should map perforators with dedicated images for any anatomical variations or defects. BARD listed three images: perforator angiograms, 3D reconstructions, and venous images, despite the latter not being routinely utilized. Unfortunately, BARD overlooked the importance of MIP images in displaying the perforator intramuscular course and repeated the same inaccurate references from its previous responses. BingAI proffered five factors, including MIPs, curved planar reconstruction (CPR), MPR, and volume-rendered images of the anterior abdominal wall. Although comprehensive, there was redundancy in its suggestions. Its five references remain unchanged from previous responses, and it included CT scan images for a clearer understanding of preoperative views. ChatGPT provided an overview of necessary images, albeit without much depth. Its citation performance improved, with three out of five references aligning with the literature. Yet, one pertained to 3D printing rather than 3D image reconstruction. Perplexity responded uniquely, providing the cited references before elaborating on them. Its answer covered four key considerations, which were delineated by the previous LLMs. Perplexity does not suggest any definitive images that should be generated during the reporting process. Despite the validity of all references, Perplexity proffers merely four unique citations, with the fifth being a duplication of reference two. Consistent with its informative approach, it concludes by offering supplementary reading.

### Prompt 4

Prompt four (Supplementary Files 1-4) assessed the LLMs’ capabilities in citing evidence and identifying the benefits of CTAs. BARD displayed multiple errors, including inaccurately labeling CTA as safer for patients with renal impairment despite using nephrotoxic iodinated contrast and wrongly claiming its superior spatial resolution over MRAs. CTAs are also more invasive than other modalities, such as Doppler Ultrasound.^16^ BARD's citations remained unverifiable. BingAI correctly suggested benefits like reduced operative time, increased availability, and cost-effectiveness. It also incorrectly stated that preoperative CTA reduces operative complications when, actually, there is no significant impact on postoperative complications, the only benefits being faster flap harvest time and shorter operative duration.^17^ It also suggested that CTA boasts higher sensitivities and specificities in perforator localization, yet studies have shown relative equivalence with MRA.^18^^,^^19^ Its references remain identical to its previous responses. ChatGPT proposed similar benefits to BingAI, albeit less detailed. Again, it suggests that CTA reduces donor site morbidity and potentially boosts flap viability, which is not completely substantiated by recent studies, displaying limitations in ChatGPT's ability to access data past 2021*.* Among its five references, three are valid, but references 1 and 4 have erroneous publication dates. Again, Perplexity first enumerates references before expanding on them. It accurately discusses benefits, namely reduced operative time, increased efficiency, precise perforator localization, and high congruence with intraoperative findings. The references are verifiable, further substantiating the presented information.

### Prompt 5

The fifth prompt (Supplementary Files 1-4) aimed to evaluate the LLMs’ competencies in discerning potential obstacles and proposing mitigation strategies during the reporting process. BARD identified three challenges: poor image quality, complex anatomy, and unexpected coexisting conditions that could complicate structural identification or cause logistical issues. Its five references remained unchanged and unverifiable. BingAI's response demonstrated mixed quality. Although its concern over patient exposure to radiation and contrast is valid, it overlooked the authors’ focus on challenges encountered during the reporting process. It subsequently addressed the issues of identifying variable perforators and angiosomes and inconsistent nomenclature, offering solutions to each problem. All its references were corroborated in the existing literature. ChatGPT offered five points regarding technical issues like image quality and surgeon communication, and anatomical challenges, including complexity, variations, and coexisting pathologies like atherosclerosis impeding interpretation. Only two of its references were verifiable, with the first being misdated. Perplexity outlined five surgical challenges with solutions. Unfortunately, the first point ambiguously discussed the problems surgeons face without CTAs. The cited study indicates that this uncertainty leads surgeons to harvest more perforators.^11^^,^^20^^,^^21^ The second point was better elucidated but omitted the study's findings of improved donor site morbidity and increased flap survival.^10^ Despite effectively pinpointing possible operative complications, it did not discuss reporting process challenges.

### Prompt 6

The final query (Supplementary Files 1-4) assessed the LLMs’ capabilities in predicting future advancements of CTA for DIEP procedures. BARD pinpointed four aspects: enhanced spatial resolution, 3D reconstructions, decreased radiation exposure, and superior contrast agents, detailing how each could impact DIEP flap procedures. Again, it provided inaccurate references. BingAI discussed one less potential area than BARD but predominantly emphasized AI technologies, like AI and ML algorithms, 3D printing, and Augmented Reality technologies, showing a deeper grasp of the potential technological future of radiology. References 2 through 6 were, unfortunately, untraceable in the literature. ChatGPT showed good insight, outlining the most potential enhancements. Perplexity identified five prospective research avenues, including real-time imaging and minimally invasive imaging technologies. Notably, it underscored the unpredictable trajectory of these advancements. Perplexity then encourages ongoing research to enhance CTA implementation into preoperative DIEP flap procedures. The six academic sources it cited were confirmed with the current literature, bolstering its discussion.

### Readability and reliability

As per Table 3, BingAI and Perplexity performed equally well in readability and reliability. BingAI had the highest Flesch Reading Ease and DISCERN scores, whereas Perplexity topped the Flesch-Kincaid Grade and Coleman-Liau Index scores. ChatGPT and BARD exhibited comparable performances. However, BARD exhibited a marginally superior Flesch Reading Ease score, whereas ChatGPT prevailed in the Flesch-Kincaid Grade level, the Coleman-Liau Index, and DISCERN scores. The t-test yielded mostly statistically significant results (P<0.05). BingAI significantly surpassed ChatGPT and Perplexity in Flesch Reading Ease scores but was like BARD. Perplexity notably exceeded other LLMs in the Flesch-Kincaid Grade and Coleman-Liau Index scores. Similarly, BingAI and Perplexity revealed significantly higher DISCERN scores than ChatGPT and BARD but were comparable to each other.

**Table 3:** Readability and reliability of LLMs' responses.

		Readability			Suitability
	Prompts	Flesch Reading Ease Score	Flesch-Kincaid Grade Level	The Coleman-Liau Index	DISCERN score
**ChatGPT**	Steps in CTA reporting	34.2	11.1	12.0	45.0
	Anatomical structures	24.6	13.2	13.0	47.0
	Important images	27.8	12.4	13.0	44.0
	Evidence and Benefits	16.2	13.3	15.0	47.0
	Potential challenges	23.5	12.5	14.0	50.0
	Future advances	21.7	14.4	15.0	43.0
**Mean(SD)**		24.7 (±6.1)	12.8 (±1.1)	13.7 (±1.2)	46.0 (±2.5)
**BARD**	Steps in CTA reporting	39.5	9.5	11.0	40.0
	Anatomical structures	28.6	11.2	11.0	32.0
	Important images	30.2	11.0	11.0	40.0
	Evidence and Benefits	38.2	10.5	11.0	39.0
	Potential challenges	37.3	10.5	11.0	51.0
	Future advances	30.7	11.9	12.0	50.0
Mean(SD)		34.1 (±4.8)	10.8 (±0.8)	11.2 (±0.4)	42.0 (±7.2)
**BingAI**	Steps in CTA reporting	34.9	12.9	12.0	63.0
	Anatomical structures	43.8	11.2	10.0	63.0
	Important images	32.1	13.5	11.0	55.0
	Evidence and Benefits	27.3	15.1	11.0	56.0
	Potential challenges	33.6	13.1	10.0	64.0
	Future advances	36.6	12.3	10.0	62.0
Mean(SD)		34.7 (±5.4)	13.0 (±1.3)	10.6 (±0.8)	60.5 (±3.9)
**Perplexity**	Steps in CTA reporting	9.1	17.0	17.0	62.0
	Anatomical structures	12.4	17.3	15.0	57.0
	Important images	9.1	21.9	18.0	56.0
	Evidence and Benefits	7.7	21.6	19.0	53.0
	Potential challenges	20.1	23.4	19.0	55.0
	Future advances	10.4	21.6	19.0	64.0
Mean(SD)		11.5 (±4.5)	20.5 (±2.7)	17.8 (±1.6)	57.8 (±4.3)

## Discussion

CTA is the gold standard imaging modality for preoperative planning of DIEP flaps to assess vital vascular anatomy.^22^ Traditionally, the reporting process is taught by specialized educators, with trainees requiring consistent feedback and supervision. The recent introduction of AI has considerably impacted medical education by augmenting traditional learning methods.^23^, ^24^, ^25^, ^26^, ^27^, ^28^ ChatGPT, BARD, and BingAI are AI chatbots with distinct underlying technologies. ChatGPT is based on the Generative Pretrained Transformer architecture, allowing it to generate human-like text in response to most inputs. BARD, on the other hand, utilizes Google's LaMDA (Language Model for Dialogue Applications) technology, which is designed to facilitate more natural and engaging conversations. BingAI is powered by OpenAI's GPT technology, but it also has the ability to provide additional multimedia content and sources for its answers.^29^^,^^30^

AI-powered tools offer students vast knowledge resources, facilitating more efficient learning. They enable interactions with virtual patients, improve comprehension of various diseases, and experiment with different treatments in a risk-free environment.^1^^,^^2^^,^^31^^,^^32^ Similarly, AI can assist in teaching complex medical skills like radiological interpretation. Reporting CTA scans of the DIEA demands an intricate understanding of complex anatomy, which can be challenging for trainees to learn and time-consuming for educators to teach. AI can circumvent these issues by providing an extensive yet comprehensible database of information, enabling trainees to learn more quickly and hone their skills, thereby supplementing traditional teaching methods. However, the effectiveness, accuracy, and safety of AI must be carefully assessed before widespread implementation in medical education.

Moreover, AI models also hold the potential to significantly improve the interpretation of CTA scans, with ML models being developed that can automatically localize DIEA perforators from CTA images. Given that CTA images are traditionally reported by radiologists/surgeons and can often be quite time-consuming, the development of this technology would allow for significantly increased efficiency. Furthermore, the determination of perforator size is typically a relatively subjective assessment, differing between the observers. Thus, a consistent, trained AI model could also provide improved accuracy and reliability in perforator assessment. This would have implications for surgeons when deciding on which perforator or hemi-abdomen to utilize intraoperatively. Overall, AI is poised to alter the landscape of preoperative planning in breast reconstruction, offering exciting potential for innovation in this field.

The present study revealed that BingAI consistently surpassed the other models in delivering precise and detailed educational responses on reporting CTA scans for preoperative planning of DIEP flaps, supported by credible references. It demonstrated superior summaries of the essential anatomical structures, surgical procedures, and potential obstacles in interpreting CTA scans for DIEP flap surgery. Additionally, it excelled in the readability and reliability tests, rivaled closely only by Perplexity (Table 2). However, its performance had limitations, such as overlooking key steps involved in reporting CTA scans of the DIEA and inaccurately suggesting that CTA may improve postoperative complications. Perplexity performed similarly to BingAI, furnishing more useful, readable information than ChatGPT and BARD, albeit not to the standard of BingAI. Its responses to prompts 1, 2, and 6 offered accurate and helpful information that a trainee could use, yet, in its other responses, it conducted more of a literature review, listing and briefly explaining studies. Overall, BARD and ChatGPT generated several incorrect responses and consistently inaccurate references.

Regarding the readability and comprehensibility results, BingAI and BARD exhibited the highest Flesch Reading Ease Scores, being statistically more significant than the other LLMs but not to each other. Statistically, Perplexity significantly exhibited the highest Flesch-Kincaid Grade level and Coleman-Liau Index scores. Overall, Perplexity and BingAI performed the best on the readability and reliability tests. Nevertheless, the authors propose that additional comparative studies involving these LLMs in relation to CTA and DIEP flaps be undertaken to acquire more robust and replicable results.^33^

The performance of the other LLMs exhibited poorer depth and accuracy when compared with BingAI, displaying greater inaccuracies on numerous prompts. For instance, BARD lacked discussion of important procedural steps and anatomical structures in response to prompts 1 and 2. Furthermore, it described incorrect images to produce and displayed a poor compilation of the utility of CTA in response to prompts 3 and 4, respectively. ChatGPT was more accurate than BARD in its responses, yet often lacked the most depth. Additionally, it suffers from limited knowledge before September 2021, significantly constraining its ability to provide updated knowledge. Perplexity offered more accurate responses than BARD and ChatGPT but was less accurate (particularly to prompts 3, 4, and 5) and lacked the depth observed in BingAI. Moreover, the references cited by Perplexity were almost always accurate and verifiable, apart from an error in recycling one reference for prompt 3. In contrast, despite possessing internet access, BARD cited erroneous references more frequently than ChatGPT, negatively impacting its reliability and DISCERN score. One of the major issues with LLMs is the reliability of their output, and BARD's frequent provision of irrelevant and even “created” references, termed “hallucination” in AI parlance, raises significant concerns.^34^ Presenting misleading information is arguably more detrimental than blatantly incorrect ones, as researchers may unknowingly propagate incorrect or even fictitious findings.

This study had several limitations. First, two plastic surgeons with extensive reporting of CTA scans evaluated the LLMs’ responses. Although their expertise maintains the assessment's credibility, there may still be elements of subjectivity. Secondly, the study focused specifically on CTA reporting for DIEP flaps, which may limit its generalizability to other medical specialties or procedures. Tailored approaches may be required when selecting the most appropriate LLM for different medical contexts, including radiology education.^35^

Therefore, further research should explore the efficacy of LLMs in various medical specialties and procedures to gain a broader understanding of their potential in medical education. Lastly, only the first response from the LLMs was considered, which allowed for a consistent comparison between the included AI models. However, it does not offer the LLMs the opportunity to provide more refined responses to follow-up questions.

## Conclusion

This study highlights the current knowledge of AI models and ML in interpreting CTAs for preoperative planning of DIEP flaps, noting multiple inaccuracies and advocating rigorous assessment of the results and references. It remains essential to acknowledge that current LLMs should not substitute medical expertise. Despite this, AI could provide a valuable adjunct to traditional trainee education in CTA reporting for preoperative planning of DIEP flaps. Furthermore, as technology advances, AI could facilitate automatic perforator identification, improving the efficiency, accuracy, and reliability of generated reports.

## Appendix Supplementary materials

Image, application 1

Image, application 2

Image, application 3

Image, application 4

Image, application 5
