# Supplementary figures and images for: Evaluating Artificial Intelligence's Role in Teaching the Reporting and Interpretation of Computed Tomographic Angiography for Preoperative Planning of the Deep Inferior Epigastric Artery Perforator Flap
Source: JPRAS Open. 2024 Apr 5;40:273–85. doi: 10.1016/j.jpra.2024.03.010 (PMC11067004; doi:10.1016/j.jpra.2024.03.010)

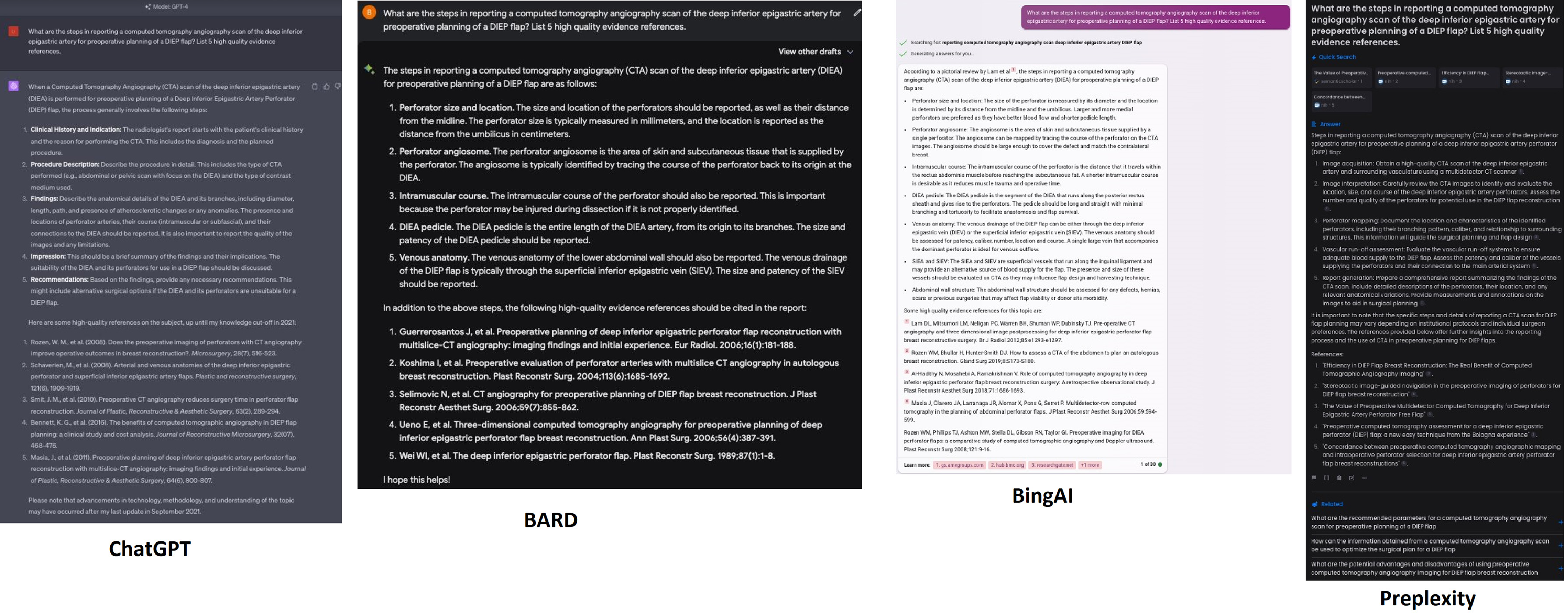

Supplement: Supplementary file 1 [file mmc1.jpg]
