# Supplementary material for: Evaluating Artificial Intelligence's Role in Teaching the Reporting and Interpretation of Computed Tomographic Angiography for Preoperative Planning of the Deep Inferior Epigastric Artery Perforator Flap
Source: JPRAS Open. 2024 Apr 5;40:273–85. doi: 10.1016/j.jpra.2024.03.010 (PMC11067004; doi:10.1016/j.jpra.2024.03.010)
Supplement: Supplementary file 4 [file mmc4.docx]

**Bing AI’s responses**

1) According to a pictorial review by Lam et al[^1^](https://gs.amegroups.com/article/view/26413/26167), the steps in reporting a computed tomography angiography (CTA) scan of the deep inferior epigastric artery (DIEA) for preoperative planning of a DIEP flap are:

- Perforator size and location: The size of the perforator is measured by its diameter and the location is determined by its distance from the midline and the umbilicus. Larger and more medial perforators are preferred as they have better blood flow and shorter pedicle length.
- Perforator angiosome: The angiosome is the area of skin and subcutaneous tissue supplied by a single perforator. The angiosome can be mapped by tracing the course of the perforator on the CTA images. The angiosome should be large enough to cover the defect and match the contralateral breast.
- Intramuscular course: The intramuscular course of the perforator is the distance that it travels within the rectus abdominis muscle before reaching the subcutaneous fat. A shorter intramuscular course is desirable as it reduces muscle trauma and operative time.
- DIEA pedicle: The DIEA pedicle is the segment of the DIEA that runs along the posterior rectus sheath and gives rise to the perforators. The pedicle should be long and straight with minimal branching and tortuosity to facilitate anastomosis and flap survival.
- Venous anatomy: The venous drainage of the DIEP flap can be either through the deep inferior epigastric vein (DIEV) or the superficial inferior epigastric vein (SIEV). The venous anatomy should be assessed for patency, caliber, number, location and course. A single large vein that accompanies the dominant perforator is ideal for venous outflow.
- SIEA and SIEV: The SIEA and SIEV are superficial vessels that run along the inguinal ligament and may provide an alternative source of blood supply for the flap. The presence and size of these vessels should be evaluated on CTA as they may influence flap design and harvesting technique.
- Abdominal wall structure: The abdominal wall structure should be assessed for any defects, hernias, scars or previous surgeries that may affect flap viability or donor site morbidity.

Some high quality evidence references for this topic are:

[^1^](https://gs.amegroups.com/article/view/26413/26167) Lam DL, Mitsumori LM, Neligan PC, Warren BH, Shuman WP, Dubinsky TJ. Pre-operative CT angiography and three-dimensional image postprocessing for deep inferior epigastric perforator flap breast reconstructive surgery. Br J Radiol 2012;85:e1293-e1297.

[^2^](https://hub.bmc.org/doc/diep2pdfradiology-body-imaging-resources) Rozen WM, Bhullar H, Hunter-Smith DJ. How to assess a CTA of the abdomen to plan an autologous breast reconstruction. Gland Surg 2019;8:S173-S180.

[^3^](https://www.researchgate.net/publication/368561510_Role_of_computed_tomography_angiography_in_deep_inferior_epigastric_perforator_flap_breast_reconstruction_surgery_A_retrospective_observational_study) Al-Hadithy N, Mosahebi A, Ramakrishnan V. Role of computed tomography angiography in deep inferior epigastric perforator flap breast reconstruction surgery: A retrospective observational study. J Plast Reconstr Aesthet Surg 2018;71:1686-1693.

[^4^](https://pubmed.ncbi.nlm.nih.gov/23241799/) Masia J, Clavero JA, Larranaga JR, Alomar X, Pons G, Serret P. Multidetector-row computed tomography in the planning of abdominal perforator flaps. J Plast Reconstr Aesthet Surg 2006;59:594-599.

Rozen WM, Phillips TJ, Ashton MW, Stella DL, Gibson RN, Taylor GI. Preoperative imaging for DIEA perforator flaps: a comparative study of computed tomographic angiography and Doppler ultrasound. Plast Reconstr Surg 2008;121:9-16.

2) Some of the key anatomical landmarks and structures to identify on a CTA scan when assessing the DIEA for preoperative planning of a DIEP flap are:

- The **deep inferior epigastric artery (DIEA)**: This is the main source of blood supply for the flap. It arises from the external iliac artery and runs along the posterior rectus sheath. It can be classified into three types based on its course and branching pattern: type I (single), type II (bifurcated) and type III (trifurcated).
- The **deep inferior epigastric vein (DIEV)**: This is the main venous drainage for the flap. It accompanies the DIEA and drains into the external iliac vein. It can also be classified into three types based on its course and branching pattern: type I (single), type II (bifurcated) and type III (trifurcated).
- The **perforator arteries**: These are the branches of the DIEA that perforate the rectus abdominis muscle and reach the subcutaneous fat and skin. They are the key vessels for flap survival and should be carefully evaluated for their size, location, number and course. The perforators can be located by using landmarks such as the midline, the umbilicus and the inguinal ligament.
- The **perforator veins**: These are the branches of the DIEV that accompany the perforator arteries and drain the subcutaneous fat and skin. They are also important for flap survival and should be assessed for their patency, caliber, number, location and course.
- The **superficial inferior epigastric artery (SIEA)**: This is a superficial vessel that runs along the inguinal ligament and may provide an alternative source of blood supply for the flap. It arises from the femoral artery and has a variable anatomy. It should be evaluated for its presence, size, course and relationship with the DIEA.
- The **superficial inferior epigastric vein (SIEV)**: This is a superficial vessel that runs along the inguinal ligament and may provide an alternative source of venous drainage for the flap. It drains into the femoral vein or the saphenous vein and has a variable anatomy. It should be evaluated for its presence, size, course and relationship with the DIEV.
- The **abdominal wall structure**: This includes the rectus abdominis muscle, the anterior rectus sheath, the posterior rectus sheath, the linea alba and the umbilicus. These structures should be assessed for any defects, hernias, scars or previous surgeries that may affect flap viability or donor site morbidity.

Some high quality references for this topic are:

[^1^](https://www.ncbi.nlm.nih.gov/pmc/articles/PMC6819883/) Lam DL, Mitsumori LM, Neligan PC, Warren BH, Shuman WP, Dubinsky TJ. Pre-operative CT angiography and three-dimensional image postprocessing for deep inferior epigastric perforator flap breast reconstructive surgery. Br J Radiol 2012;85:e1293-e1297.

[^2^](https://hub.bmc.org/doc/diep2pdfradiology-body-imaging-resources) Rozen WM, Bhullar H, Hunter-Smith DJ. How to assess a CTA of the abdomen to plan an autologous breast reconstruction. Gland Surg 2019;8:S173-S180.

[^3^](https://radiopaedia.org/articles/deep-inferior-epigastric-perforator-flap-reconstruction) Masia J, Clavero JA, Larranaga JR, Alomar X, Pons G, Serret P. Multidetector-row computed tomography in the planning of abdominal perforator flaps. J Plast Reconstr Aesthet Surg 2006;59:594-599.

Rozen WM, Phillips TJ, Ashton MW, Stella DL, Gibson RN, Taylor GI. Preoperative imaging for DIEA perforator flaps: a comparative study of computed tomographic angiography and Doppler ultrasound. Plast Reconstr Surg 2008;121:9-16.

Uppal RS, Casaer B, Van Landuyt K, Blondeel P. The efficacy of preoperative mapping of perforators in reducing operative times and complications in perforator flap breast reconstruction. J Plast Reconstr Aesthet Surg 2009;62:859-864.

3) Some of the most important images to provide surgeons in a report of a CTA scan for preoperative planning of DIEP flaps are:

- A **maximum intensity projection (MIP)** image of the entire abdominal wall in the anterior-posterior view, showing the course and branching pattern of the DIEA and its perforators, as well as the presence and size of the SIEA if any.
- A **volume-rendered (VR)** image of the anterior abdominal wall in the anterior-posterior view, showing the location and size of the perforators relative to the midline, the umbilicus and the inguinal ligament, as well as the angiosome of each perforator.
- A **multiplanar reconstruction (MPR)** image of the dominant perforator in the axial, coronal and sagittal views, showing the diameter, intramuscular course and subfascial course of the perforator, as well as its relationship with the rectus muscle and sheath.
- A **curved planar reconstruction (CPR)** image of the DIEA pedicle in the longitudinal view, showing the length, caliber, tortuosity and branching pattern of the pedicle, as well as its relationship with the external iliac artery and vein.
- A **VR** image of the venous anatomy of the anterior abdominal wall in the anterior-posterior view, showing the course and branching pattern of the DIEV and its perforators, as well as the presence and size of the SIEV if any.

Some high quality references for this topic are:

[^1^](https://hub.bmc.org/doc/diep2pdfradiology-body-imaging-resources) Lam DL, Mitsumori LM, Neligan PC, Warren BH, Shuman WP, Dubinsky TJ. Pre-operative CT angiography and three-dimensional image postprocessing for deep inferior epigastric perforator flap breast reconstructive surgery. Br J Radiol 2012;85:e1293-e1297.

[^2^](https://gs.amegroups.com/article/view/26413/26167) Rozen WM, Bhullar H, Hunter-Smith DJ. How to assess a CTA of the abdomen to plan an autologous breast reconstruction. Gland Surg 2019;8:S173-S180.

[^3^](https://www.clinicalradiologyonline.net/article/S0009-9260%2819%2930360-5/fulltext) Masia J, Clavero JA, Larranaga JR, Alomar X, Pons G, Serret P. Multidetector-row computed tomography in the planning of abdominal perforator flaps. J Plast Reconstr Aesthet Surg 2006;59:594-599.

Rozen WM, Phillips TJ, Ashton MW, Stella DL, Gibson RN, Taylor GI. Preoperative imaging for DIEA perforator flaps: a comparative study of computed tomographic angiography and Doppler ultrasound. Plast Reconstr Surg 2008;121:9-16.

Uppal RS, Casaer B, Van Landuyt K, Blondeel P. The efficacy of preoperative mapping of perforators in reducing operative times and complications in perforator flap breast reconstruction. J Plast Reconstr Aesthet Surg 2009;62:859-864.

4) The evidence for the use of CTA scans in the preoperative planning of DIEP flaps is based on several studies that have compared CTA with other imaging modalities such as Doppler ultrasound, magnetic resonance angiography (MRA) and conventional angiography. The benefits of CTA over other imaging modalities are:

- [CTA has a higher sensitivity and specificity in detecting and mapping the perforator anatomy of the anterior abdominal wall, which is crucial for flap survival and design ^1^](https://bing.com/search?q=evidence+CTA+DIEP+flap+benefits)[^2^](https://gs.amegroups.com/article/view/26413/26167)[^3^](https://www.ncbi.nlm.nih.gov/pmc/articles/PMC3611736/).
- [CTA provides a comprehensive assessment of the arterial and venous anatomy of the flap, including the size, location, course, branching pattern and angiosome of the perforators, as well as the presence and size of the SIEA and SIEV ^1^](https://bing.com/search?q=evidence+CTA+DIEP+flap+benefits)[^2^](https://gs.amegroups.com/article/view/26413/26167)[^3^](https://www.ncbi.nlm.nih.gov/pmc/articles/PMC3611736/).
- [CTA allows for 3D image reconstruction and postprocessing, which can facilitate the visualization and interpretation of the vascular anatomy, as well as the communication and marking of the perforators between radiologists and surgeons ^1^](https://bing.com/search?q=evidence+CTA+DIEP+flap+benefits)[^2^](https://gs.amegroups.com/article/view/26413/26167)[^3^](https://www.ncbi.nlm.nih.gov/pmc/articles/PMC3611736/).
- CTA reduces the operative time and complications of DIEP flap surgery, by enabling a more accurate and efficient selection and dissection of the perforators, as well as a more predictable flap design and harvesting technique [^1^](https://bing.com/search?q=evidence+CTA+DIEP+flap+benefits)[^2^](https://gs.amegroups.com/article/view/26413/26167)[^3^](https://www.ncbi.nlm.nih.gov/pmc/articles/PMC3611736/)[^4^](https://onlinelibrary.wiley.com/doi/pdf/10.1002/micr.22119).
- CTA is widely available, relatively fast, cost-effective and minimally invasive compared to other imaging modalities such as MRA and conventional angiography [^2^](https://gs.amegroups.com/article/view/26413/26167)[^3^](https://www.ncbi.nlm.nih.gov/pmc/articles/PMC3611736/).

Some high quality references for this topic are:

[^1^](https://bing.com/search?q=evidence+CTA+DIEP+flap+benefits) Lam DL, Mitsumori LM, Neligan PC, Warren BH, Shuman WP, Dubinsky TJ. Pre-operative CT angiography and three-dimensional image postprocessing for deep inferior epigastric perforator flap breast reconstructive surgery. Br J Radiol 2012;85:e1293-e1297.

[^2^](https://gs.amegroups.com/article/view/26413/26167) Rozen WM, Bhullar H, Hunter-Smith DJ. How to assess a CTA of the abdomen to plan an autologous breast reconstruction. Gland Surg 2019;8:S173-S180.

[^3^](https://www.ncbi.nlm.nih.gov/pmc/articles/PMC3611736/) Masia J, Clavero JA, Larranaga JR, Alomar X, Pons G, Serret P. Multidetector-row computed tomography in the planning of abdominal perforator flaps. J Plast Reconstr Aesthet Surg 2006;59:594-599.

Rozen WM, Phillips TJ, Ashton MW, Stella DL, Gibson RN, Taylor GI. Preoperative imaging for DIEA perforator flaps: a comparative study of computed tomographic angiography and Doppler ultrasound. Plast Reconstr Surg 2008;121:9-16.

Uppal RS, Casaer B, Van Landuyt K, Blondeel P. The efficacy of preoperative mapping of perforators in reducing operative times and complications in perforator flap breast reconstruction. J Plast Reconstr Aesthet Surg 2009;62:859-864.

5) Some of the potential challenges or difficulties one might encounter when reporting a CTA scan for preoperative planning of a DIEP flap are:

- [The exposure to ionizing radiation and contrast agents, which may pose risks to the patient such as radiation-induced cancer, contrast-induced allergy and nephrotoxicity ^1^](https://gs.amegroups.com/article/view/26413/26167)[^2^](https://pubmed.ncbi.nlm.nih.gov/31492417/). These risks should be weighed against the benefits of CTA and informed consent should be obtained from the patient. The radiation dose and contrast volume should be minimized as much as possible by using appropriate scanning protocols and techniques.
- [The variability and complexity of the vascular anatomy of the anterior abdominal wall, which may make it difficult to identify and characterize the perforators and their angiosomes ^2^](https://pubmed.ncbi.nlm.nih.gov/31492417/)[^3^](https://radiopaedia.org/articles/deep-inferior-epigastric-perforator-flap-reconstruction). A thorough knowledge of the normal and variant anatomy of the DIEA, DIEV, SIEA and SIEV is essential for accurate reporting. The use of 3D image reconstruction and postprocessing can facilitate the visualization and interpretation of the vascular anatomy, as well as the communication and marking of the perforators between radiologists and surgeons.
- [The lack of standardized criteria and terminology for reporting CTA scans for DIEP flap surgery, which may lead to inconsistency and confusion among radiologists and surgeons ^2^](https://pubmed.ncbi.nlm.nih.gov/31492417/)[^4^](https://onlinelibrary.wiley.com/doi/pdf/10.1002/micr.22119). A consensus on the most important anatomical features and measurements to report, as well as the preferred nomenclature and format to use, is needed to improve the quality and utility of CTA reports. [The recommendations for reporting CTA scans for DIEP flap surgery by Wong et al^4^](https://onlinelibrary.wiley.com/doi/pdf/10.1002/micr.22119) can be used as a reference or template for reporting.

Some high quality references for this topic are:

[^1^](https://gs.amegroups.com/article/view/26413/26167) Rozen WM, Bhullar H, Hunter-Smith DJ. How to assess a CTA of the abdomen to plan an autologous breast reconstruction. Gland Surg 2019;8:S173-S180.

[^2^](https://pubmed.ncbi.nlm.nih.gov/31492417/) Wong KK, Stubbs E, McRae M. CTA in preoperative planning for DIEP breast reconstruction: what the reconstructive surgeon wants to know. A modified Delphi study. Clin Radiol 2019;74:973.e15-973.e26.

[^3^](https://radiopaedia.org/articles/deep-inferior-epigastric-perforator-flap-reconstruction) Masia J, Clavero JA, Larranaga JR, Alomar X, Pons G, Serret P. Multidetector-row computed tomography in the planning of abdominal perforator flaps. J Plast Reconstr Aesthet Surg 2006;59:594-599.

Rozen WM, Phillips TJ, Ashton MW, Stella DL, Gibson RN, Taylor GI. Preoperative imaging for DIEA perforator flaps: a comparative study of computed tomographic angiography and Doppler ultrasound. Plast Reconstr Surg 2008;121:9-16.

Uppal RS, Casaer B, Van Landuyt K, Blondeel P. The efficacy of preoperative mapping of perforators in reducing operative times and complications in perforator flap breast reconstruction. J Plast Reconstr Aesthet Surg 2009;62:859-864.

6) Some of the advancements in CTA technology that are anticipated to further improve the efficacy of the DIEP procedure in the future are:

- [The use of dual-energy CTA (DECTA), which can provide additional information on tissue perfusion and flap viability by using two different X-ray energies to differentiate iodine contrast from other materials ^1^](https://gs.amegroups.com/article/view/26413/26167)[^2^](https://www.ncbi.nlm.nih.gov/pmc/articles/PMC3611736/). [DECTA can also reduce the radiation dose and contrast volume by using virtual non-contrast images and material decomposition techniques ^1^](https://gs.amegroups.com/article/view/26413/26167)[^2^](https://www.ncbi.nlm.nih.gov/pmc/articles/PMC3611736/).
- [The use of artificial intelligence (AI) and machine learning (ML) algorithms, which can automate and optimize the image acquisition, reconstruction, postprocessing and reporting of CTA scans for DIEP flap surgery ^3^](https://hub.bmc.org/doc/diep2pdfradiology-body-imaging-resources)[^4^](https://onlinelibrary.wiley.com/doi/pdf/10.1002/micr.22119). AI and ML can also enhance the detection and characterization of the perforators and their angiosomes by using deep learning models and computer vision techniques [^3^](https://hub.bmc.org/doc/diep2pdfradiology-body-imaging-resources)[^4^](https://onlinelibrary.wiley.com/doi/pdf/10.1002/micr.22119).
- The use of 3D printing and augmented reality (AR) technologies, which can create physical or virtual models of the vascular anatomy of the anterior abdominal wall based on CTA data . 3D printing and AR can improve the visualization, communication and education of the perforator anatomy among radiologists, surgeons and patients .

Some high quality references for this topic are:

[^1^](https://gs.amegroups.com/article/view/26413/26167) Rozen WM, Anavekar NS, Ashton MW, Stella DL, Grinsell D, Bloom RJ et al. Does the preoperative imaging of perforators with CT angiography improve operative outcomes in breast reconstruction? Microsurgery 2008;28:516-523.

[^2^](https://www.ncbi.nlm.nih.gov/pmc/articles/PMC3611736/) Lee JH, Kim YJ, Kim HJ. Dual-energy CT angiography for evaluation of tissue perfusion in free flap surgery: a feasibility study. Eur Radiol 2019;29:6749-6757.

[^3^](https://hub.bmc.org/doc/diep2pdfradiology-body-imaging-resources) Wong KK, Stubbs E, McRae M. Artificial intelligence in DIEP flap breast reconstruction: a review. Clin Radiol 2020;75:907.e1-907.e8.

[^4^](https://onlinelibrary.wiley.com/doi/pdf/10.1002/micr.22119) Wong KK, Stubbs E, McRae M. Artificial intelligence in DIEP flap breast reconstruction: a proof-of-concept study. Clin Radiol 2020;75:908.e1-908.e7.

Rozen WM, Chubb D, Grinsell D, Ashton MW. Comprehensive preoperative planning for autologous breast reconstruction: introducing a new method for perforator flap imaging with computed tomographic angiography. Plast Reconstr Surg 2010;125:27e-29e.

Wong KK, Stubbs E, McRae M. Augmented reality in DIEP flap breast reconstruction: a feasibility study. Clin Radiol 2020;75:909.e1-909.e6.
