# Supplementary material for: Evaluating Artificial Intelligence's Role in Teaching the Reporting and Interpretation of Computed Tomographic Angiography for Preoperative Planning of the Deep Inferior Epigastric Artery Perforator Flap
Source: JPRAS Open. 2024 Apr 5;40:273–85. doi: 10.1016/j.jpra.2024.03.010 (PMC11067004; doi:10.1016/j.jpra.2024.03.010)
Supplement: Supplementary file 5 [file mmc5.docx]

**Perplexity’s responses**

1) Steps in reporting a computed tomography angiography (CTA) scan of the deep inferior epigastric artery for preoperative planning of a deep inferior epigastric artery perforator (DIEP) flap:

1. Image acquisition. Obtain a high-quality CTA scan of the deep inferior epigastric artery and surrounding vasculature using a multidetector CT scanner.
2. Image interpretation. Carefully review the CTA images to identify and evaluate the location, size, and course of the deep inferior epigastric artery perforators. Assess the number and quality of the perforators for potential use in the DIEP flap reconstruction.
3. Perforator mapping. Document the location and characteristics of the identified perforators, including their branching pattern, caliber, and relationship to surrounding structures. This information will guide the surgical planning and flap design.
4. Vascular run-off assessment. Evaluate the vascular run-off systems to ensure adequate blood supply to the DIEP flap. Assess the patency and caliber of the vessels supplying the perforators and their connection to the main arterial system.
5. Report generation. Prepare a comprehensive report summarizing the findings of the CTA scan. Include detailed descriptions of the perforators, their location, and any relevant anatomical variations. Provide measurements and annotations on the images to aid in surgical planning.

It is important to note that the specific steps and details of reporting a CTA scan for DIEP flap planning may vary depending on institutional protocols and individual surgeon preferences. The references provided below offer further insights into the reporting process and the use of CTA in preoperative planning for DIEP flaps.

References:

1. "Efficiency in DIEP Flap Breast Reconstruction: The Real Benefit of Computed Tomographic Angiography Imaging"
2. "Stereotactic image-guided navigation in the preoperative imaging of perforators for DIEP flap breast reconstruction"
3. "The Value of Preoperative Multidetector Computed Tomography for Deep Inferior Epigastric Artery Perforator Free Flap"
4. "Preoperative computed tomography assessment for a deep inferior epigastric perforator (DIEP) flap new easy technique from the Bologna experience"
5. "Concordance between preoperative computed tomography angiographic mapping and intraoperative perforator selection for deep inferior epigastric artery perforator flap breast reconstructions"

2) Key anatomical landmarks and structures to identify on a computed tomography angiography (CTA) scan when assessing the deep inferior epigastric artery (DIEA) for preoperative planning of a deep inferior epigastric artery perforator (DIEP) flap include:

1. The DIEA: The DIEA is the main vascular pedicle that supplies the abdominal wall and the overlying skin and subcutaneous tissue. It gives rise to multiple perforators that can be used for DIEP flap reconstruction
2. Perforators: The perforators are small blood vessels that branch off the DIEA and penetrate the rectus abdominis muscle to supply the overlying skin and subcutaneous tissue. They are the key structures to identify on a CTA scan for DIEP flap planning
3. Vascular run-off systems: The vascular run-off systems are the arteries and veins that provide blood supply to the perforators and the DIEP flap. They include the superior and inferior epigastric arteries and veins, the deep circumflex iliac artery and vein, and the superficial circumflex iliac artery and vein
4. Abdominal wall musculature: The rectus abdominis muscle and its surrounding musculature are important landmarks to identify on a CTA scan. The perforators typically arise from the DIEA within or adjacent to the rectus abdominis muscle
5. Skin and subcutaneous tissue: The skin and subcutaneous tissue overlying the abdominal wall are also important structures to evaluate on a CTA scan. The quality and quantity of the overlying tissue can affect the size and shape of the DIEP flap that can be harvested

These references provide further insights into the key anatomical landmarks and structures to identify on a CTA scan when assessing the deep inferior epigastric artery for preoperative planning of a DIEP flap.

References:

1. "Preoperative computed tomography assessment for a deep inferior epigastric perforator (DIEP) flap new easy technique from the Bologna experience"
2. "The Value of Preoperative Multidetector Computed Tomography for Deep Inferior Epigastric Artery Perforator Free Flap"
3. "Stereotactic image-guided navigation in the preoperative imaging of perforators for DIEP flap breast reconstruction"
4. "Efficiency in DIEP Flap Breast Reconstruction. The Real Benefit of Computed Tomographic Angiography Imaging"
5. "Concordance between preoperative computed tomography angiographic mapping and intraoperative perforator selection for deep inferior epigastric artery perforator flap breast reconstructions"
6. "Planning deep inferior epigastric perforator flaps for breast reconstruction comparison between multidetector computed tomography and magnetic resonance imaging"

3) Here are five high-quality references that discuss the important images to provide surgeons in a report of a computed tomography angiography (CTA) scan for preoperative planning of DIEP flaps:

1. "The Value of Preoperative Multidetector Computed Tomography for Deep Inferior Epigastric Artery Perforator Free Flap": This report highlights the usefulness of MDCT angiography in the preoperative planning of breast reconstruction with abdominal flaps. It emphasizes the need for a preoperative evaluation of the precise location of perforating vessels and vascular run-off systems.
2. "Stereotactic image-guided navigation in the preoperative imaging of perforators for DIEP flap breast reconstruction": This study explores the feasibility of CT-guided stereotactic imaging in DIEP flap surgery. It compares findings from conventional CTA, CT-guided stereotactic imaging, and operative findings, demonstrating the accuracy of stereotactic navigation in localizing major perforators.
3. "The utility of three-dimensional models in complex microsurgical reconstruction": This article discusses the benefits of using three-dimensional (3D) models in microsurgical reconstruction. It highlights the improved visualization and exact representation of the surgical site provided by DIEP models, which can aid in preoperative planning and intraoperative dissection.
4. "Breast Reconstruction Using Patients Own Tissue Based on CT Angiography and -D Surface Scanning": This study focuses on breast reconstruction using soft tissue flaps from the abdominal region, such as the DIEP flap. It discusses the use of modern imaging technologies, including DIEP surface scanning and DIEP CTA, for optimizing preoperative planning in autologous abdominal breast reconstruction.
5. Additional reference “A novel computed tomography angiography technique guided preoperative localization and design of anterolateral thigh perforator flap": This reference describes a novel technique using CT angiography for preoperative localization and design of the anterolateral thigh perforator flap.

These references provide valuable insights into the use of CTA and other imaging techniques for preoperative planning of DIEP flaps, highlighting the importance of accurately identifying perforating vessels and vascular anatomy.

4) Here are five high-quality references that discuss the evidence for the use of computed tomography angiography (CTA) scans in the preoperative planning of deep inferior epigastric artery perforator (DIEP) flaps and the benefits it offers over other imaging modalities:

1. "Preoperative computed tomography angiography for planning DIEP flap breast reconstruction reduces operative time and overall complications": This study demonstrates the potential benefits of using preoperative CTA scans in DIEP flap breast reconstruction. It shows a significant reduction in flap harvest time and overall operative time, leading to improved efficiency and potentially fewer complications.
2. "Efficiency in DIEP Flap Breast Reconstruction: The Real Benefit of Computed Tomographic Angiography Imaging": This study compares operative times for DIEP flap harvest with and without preoperative CTA imaging. It shows that nonblinded surgeons who assessed the CTA scan preoperatively were more efficient in all categories, including time to first perforator identification, time to perforator decision-making, time to flap harvest, and total operative time.
3. "Stereotactic image-guided navigation in the preoperative imaging of perforators for DIEP flap breast reconstruction": This study explores the feasibility of using CT-guided stereotactic imaging in DIEP flap surgery. It demonstrates that CT-guided stereotactic imaging is an accurate method for preoperative planning of DIEP flaps, providing additional and potentially more accurate data compared to conventional CTA.
4. "Preoperative computed tomography assessment for a deep inferior epigastric perforator (DIEP) flaps new easy technique from the Bologna experience": This study evaluates a new preoperative CTA protocol and reconstruction method for assessing DIEP flaps. It shows high accuracy in CTA imaging assessment of the perforators, with high reproducibility between CT findings and surgical findings.
5. "Concordance between preoperative computed tomography angiographic mapping and intraoperative perforator selection for deep inferior epigastric artery perforator flap breast reconstructions"[:](https://pubmed.ncbi.nlm.nih.gov/29302477/) This study examines the concordance between preoperative CTA mapping and intraoperative perforator selection in DIEP flap breast reconstructions. It reveals a concordance rate of 67.3% between preoperative and intraoperative perforator selection, highlighting the potential usefulness of CTA in guiding surgical decision-making.

These references provide evidence for the effectiveness of CTA scans in preoperative planning of DIEP flaps, showing benefits such as reduced operative time, improved efficiency, accurate localization of perforators, and high concordance with intraoperative findings.

5) Here are five high-quality references that discuss the potential challenges or difficulties one might encounter when reporting a computed tomography angiogram (CTA) for preoperative planning of a deep inferior epigastric artery perforator (DIEP) flap, and how they can be addressed:

1. "Efficiency in DIEP Flap Breast Reconstruction: The Real Benefit of Computed Tomographic Angiography Imaging": This study highlights the importance of surgeon familiarity with preoperative CTA scans and the potential for increased efficiency in DIEP flap harvest. It suggests that surgeons who are not familiar with interpreting CTA scans may experience challenges in identifying perforators and making decisions based on the imaging.
2. "Preoperative computed tomography angiography for planning DIEP flap breast reconstruction reduces operative time and overall complications": This study identifies the potential challenge of increased operative time and complications associated with DIEP flap breast reconstruction without preoperative CTA imaging. It suggests that preoperative CTA imaging can help reduce operative time and overall complications.
3. "Stereotactic image-guided navigation in the preoperative imaging of perforators for DIEP flap breast reconstruction": This study identifies the variability of perforator anatomy as a potential challenge in preoperative imaging for DIEP flap surgery. It suggests that CT-guided stereotactic imaging can provide additional and potentially more accurate data compared to conventional CTA.
4. "Concordance between preoperative computed tomography angiographic mapping and intraoperative perforator selection for deep inferior epigastric artery perforator flap breast reconstructions": This study identifies the potential challenge of low concordance between preoperative CTA mapping and intraoperative perforator selection in DIEP flap breast reconstructions. It suggests that a standardized protocol and surgeon experience can help improve concordance.
5. "The Value of Preoperative Multidetector Computed Tomography for Deep Inferior Epigastric Artery Perforator Free Flap": This study identifies the need for a preoperative evaluation of the precise location of perforating vessels and vascular run-off systems in DIEP flap surgery. It suggests that MDCT angiography can provide a noninvasive approach to the vascular anatomy of the entire anterior abdominal wall.

These references highlight the importance of surgeon familiarity with preoperative CTA scans, the potential for increased efficiency and reduced complications with preoperative CTA imaging, the variability of perforator anatomy, low concordance between preoperative CTA mapping and intraoperative perforator selection, and the usefulness of MDCT angiography in preoperative planning of DIEP flaps.

6) Advancements in computed tomography angiography (CTA) technology that are anticipated to further improve the efficacy of the deep inferior epigastric artery perforator (DIEP) procedure in the future include:

1. Improved image resolution: Future advancements in CTA technology may lead to even higher image resolution, allowing for more precise visualization of perforators and vascular structures.
2. Three-dimensional (3D) reconstruction: The use of 3D reconstruction techniques in CTA imaging can provide surgeons with a more comprehensive and detailed view of the vascular anatomy, facilitating accurate preoperative planning of DIEP flaps.
3. Real-time imaging: The development of real-time CTA imaging techniques may enable surgeons to visualize blood flow dynamics and assess the viability of perforators during the procedure, enhancing surgical decision-making and flap outcomes.
4. Artificial intelligence (AI) and machine learning: Integration of AI algorithms and machine learning techniques into CTA analysis may assist in automated identification and segmentation of perforators, reducing the time and effort required for manual interpretation.
5. Minimally invasive imaging: Advancements in CTA technology may lead to the development of less invasive imaging techniques, such as handheld or portable CTA devices, allowing for easier and more accessible preoperative imaging for DIEP flap planning.

These advancements in CTA technology have the potential to improve the accuracy, efficiency, and outcomes of the DIEP procedure by providing surgeons with enhanced visualization and better understanding of the patient's vascular anatomy. However, it is important to note that these anticipated advancements are based on current research and technological trends, and their implementation and effectiveness in clinical practice will require further investigation and validation.

References:

1. "Efficiency in DIEP Flap Breast Reconstruction: The Real Benefit of Computed Tomographic Angiography Imaging"
2. "Preoperative computed tomography angiography for planning DIEP flap breast reconstruction reduces operative time and overall complications"
3. "Stereotactic image-guided navigation in the preoperative imaging of perforators for DIEP flap breast reconstruction"
4. "Preoperative computed tomography assessment for a deep inferior epigastric perforator (DIEP) flap: a new easy technique from the Bologna experience"
5. "Concordance between preoperative computed tomography angiographic mapping and intraoperative perforator selection for deep inferior epigastric artery perforator flap breast reconstructions"
6. "The Value of Preoperative Multidetector Computed Tomography for Deep Inferior Epigastric Artery Perforator Free Flap"
